# Determining the effects of targeted high-definition transcranial direct current stimulation on reducing post-stroke upper limb motor impairments—a randomized cross-over study

**DOI:** 10.1186/s13063-023-07886-w

**Published:** 2024-01-09

**Authors:** Rita Huan-Ting Peng, Dorothy He, Shirley A. James, Jordan N. Williamson, Carly Skadden, Sanjiv Jain, Wael Hassaneen, Amrendra Miranpuri, Amandeep Kaur, Jesus N. Sarol, Yuan Yang

**Affiliations:** 1 Department of Bioengineering, Grainger College of Engineering, University of Illinois Urbana-Champaign, Urbana, IL USA; 2 Carle Foundation Hospital, Urbana, IL USA; 3 The University of Oklahoma College of Medicine, University of Oklahoma Health Sciences Center, Oklahoma City, OK USA; 4 Department of Biostatistics and Epidemiology, Hudson College of Public Health, University of Oklahoma Health Sciences Center, Oklahoma City, OK USA; 5 Beckman Institute for Advanced Science and Technology, University of Illinois Urbana-Champaign, Urbana, IL USA; 6 Department of Physical Therapy and Human Movement Sciences, Northwestern University, Chicago, IL USA; 7 Carle Illinois College of Medicine, Urbana, IL USA; 8 Interdisciplinary Health Sciences Institute, University of Illinois Urbana-Champaign, Urbana, IL USA

**Keywords:** Targeted high-definition tDCS, Flexion synergy, Spasticity, Stroke

## Abstract

**Background:**

Stroke is one of the leading causes of death in the USA and is a major cause of serious disability for adults. This randomized crossover study examines the effect of targeted high-definition transcranial direct current transcranial brain stimulation (tDCS) on upper extremity motor recovery in patients in the post-acute phase of stroke recovery.

**Methods:**

This randomized double-blinded cross-over study includes four intervention arms: anodal, cathodal, and bilateral brain stimulation, as well as a placebo stimulation. Participants receive each intervention in a randomized order, with a 2-week washout period between each intervention. The primary outcome measure is change in Motor Evoked Potential. Secondary outcome measures include the Fugl-Meyer Upper Extremity (FM-UE) score, a subset of FM-UE (A), related to the muscle synergies, and the Modified Ashworth Scale.

**Discussion:**

We hypothesize that anodal stimulation to the ipsilesional primary motor cortex will increase the excitability of the damaged cortico-spinal tract, reducing the UE flexion synergy and enhancing UE motor function. We further hypothesize that targeted cathodal stimulation to the contralesional premotor cortex will decrease activation of the cortico-reticulospinal tract (CRST) and the expression of the upper extremity (UE) flexion synergy and spasticity. Finally, we hypothesize bilateral stimulation will achieve both results simultaneously. Results from this study could improve understanding of the mechanism behind motor impairment and recovery in stroke and perfect the targeting of tDCS as a potential stroke intervention. With the use of appropriate screening, we anticipate no ethical or safety concerns. We plan to disseminate these research results to journals related to stroke recovery, engineering, and medicine.

**Trial registration:**

ClinicalTrials.gov NCT05479006. Registered on 26 July 2022.

## Administrative information

Note: the numbers in curly brackets in this protocol refer to SPIRIT checklist item numbers. The order of the items has been modified to group similar items (see http://www.equator-network.org/reporting-guidelines/spirit-2013-statement-defining-standard-protocol-items-for-clinical-trials/).

**Table:** 

Title {1}	Determining the effects of targeted high-definition transcranial direct current stimulation (THD-tDCS) on Reducing Post-Stroke Upper Limb Motor Impairments
Trial registration {2a and 2b}.	The trial is registered with ClinicalTrials.gov. Registration number: NCT05479006
Protocol version {3}	23 July 2022, Version n. 1
Funding {4}	Research reported in this publication was supported by the American Heart Association (AHA 932980). AHA did not directly involve in the design of the study and collection, analysis, and interpretation of data or in writing the manuscript.
Author details {5a}	Rita Huan-Ting Peng, MS^1,2*^, Dorothy He, BS^3*^, Shirley A. James, PT PhD^4^, Jordan N. Williamson, MS^1^, Carly Skadden, MPH^2^, Sanjiv Jain, MD^2,7^, Wael Hassaneen, MD^2,7^, Amrendra Miranpuri, MD^2,7^, Amandeep Kaur, MPH^8^, Jesus N Sarol, PhD^8^, Yuan Yang, PhD MS^1,2,5,6^ • RHTP is a first year PhD student in Bioengineering at University of Illinois at Urbana-Champaign, Urbana, IL. • DH is a third-year medical student at the University of Oklahoma Health Sciences Center, Oklahoma City, OK. • SAJ is a research faculty member of the Hudson College of Public Health with degrees in epidemiology and physical therapy, at the University of Oklahoma Health Sciences Center, Oklahoma City, OK. • JNW is an academic hourly employee at University of Illinois at Urbana-Champaign, Urbana, IL. • CS is a Clinical Research Program Manager at the Carle Heart and Vascular Institute and Carle Neuroscience Institute of Stephens Family Clinical Research Institute at Carle Foundation Hospital, Urbana, IL. • SJ is a Clinical Associate Professor, Clinical Sciences and Course Director in Musculoskeletal at Carle Illinois College of Medicine, and a Physical Medicine and Rehabilitation physician at Carle Foundation Hospital, Urbana, IL. • WH is the Associate Medical Director for the Carle Neuroscience Institute neurosurgeon at Carle Foundation Hospital, a Clinical Associate Professor at the Carle Illinois College of Medicine, and a neurosurgeon at Carle Foundation Hospital, Urbana, IL. • AM is a Clinical Associate Professor of Clinical Sciences at Carle Illinois College of Medicine and a neurosurgeon at Carle Foundation Hospital, Urbana, IL. • YY is an Associate Professor of Bioengineering at University of Illinois at Urbana -Champaign, Urbana, IL and an Associate Professor in the Clinical Imaging Research Center at Carle Foundation Hospital, Urbana, IL. He is also an adjunct faculty in Physical Therapy and Human Movement Sciences, Interdepartmental Neuroscience Program, and Developmental Science at Northwestern University, Chicago, IL. • AK is a Research Biostatistician for the Interdisciplinary Health Sciences Institute at University of Illinois at Urbana-Champaign, Urbana, IL • JNS is a Sr. Research Biostatistician for the Interdisciplinary Health Sciences Institute at University of Illinois at Urbana-Champaign, Urbana, IL Corresponding Author: Yuan Yang (yuany@illinois.edu)
Name and contact information for the trial sponsor {5b}	Sally Shipley, MBA, CCRP Manager, Research Finance and Regulatory Affairs Carle Foundation Hospital 509 W University Ave, Urbana IL 61801 Email: sally.shipley@carle.com Phone: 217–383-6537
Role of sponsor {5c}	The study sponsor and study funders do not have any role or ultimate authority in the study design; collection, management, analysis, and interpretation of data; writing of the report; or in the decision to submit the report for publication.

## Introduction

### Background and rationale {6a}

Approximately 9.4 million Americans over the age of 20 live with the impairments associated with stroke, and 795,000 people in the USA experience a new or recurrent stroke each year. This number is expected to double by the year 2050 [1]. Impairments lead to functional limitations, which make vocational pursuit, independent living, and social interaction difficult or impossible. These facts and figures make the management of stroke impairments critically important [1].

Previous intervention for stroke rehabilitation has largely focused on functional impairments [2]; however, transcranial direct current stimulation (tDCS) is an emerging and promising intervention that can transform a patient’s remaining motor control after stroke [1]. Anodal stimulation to the lesioned hemisphere can have a beneficial effect on motor function recovery [3–7]. Cathodal stimulation to the non-lesioned hemisphere can also lead to improvement in upper extremity motor function for individuals in the post-acute phase of stroke recovery. To date, the mechanism and ideal target for this stimulation remains unclear [8].

**Fig. 1:**
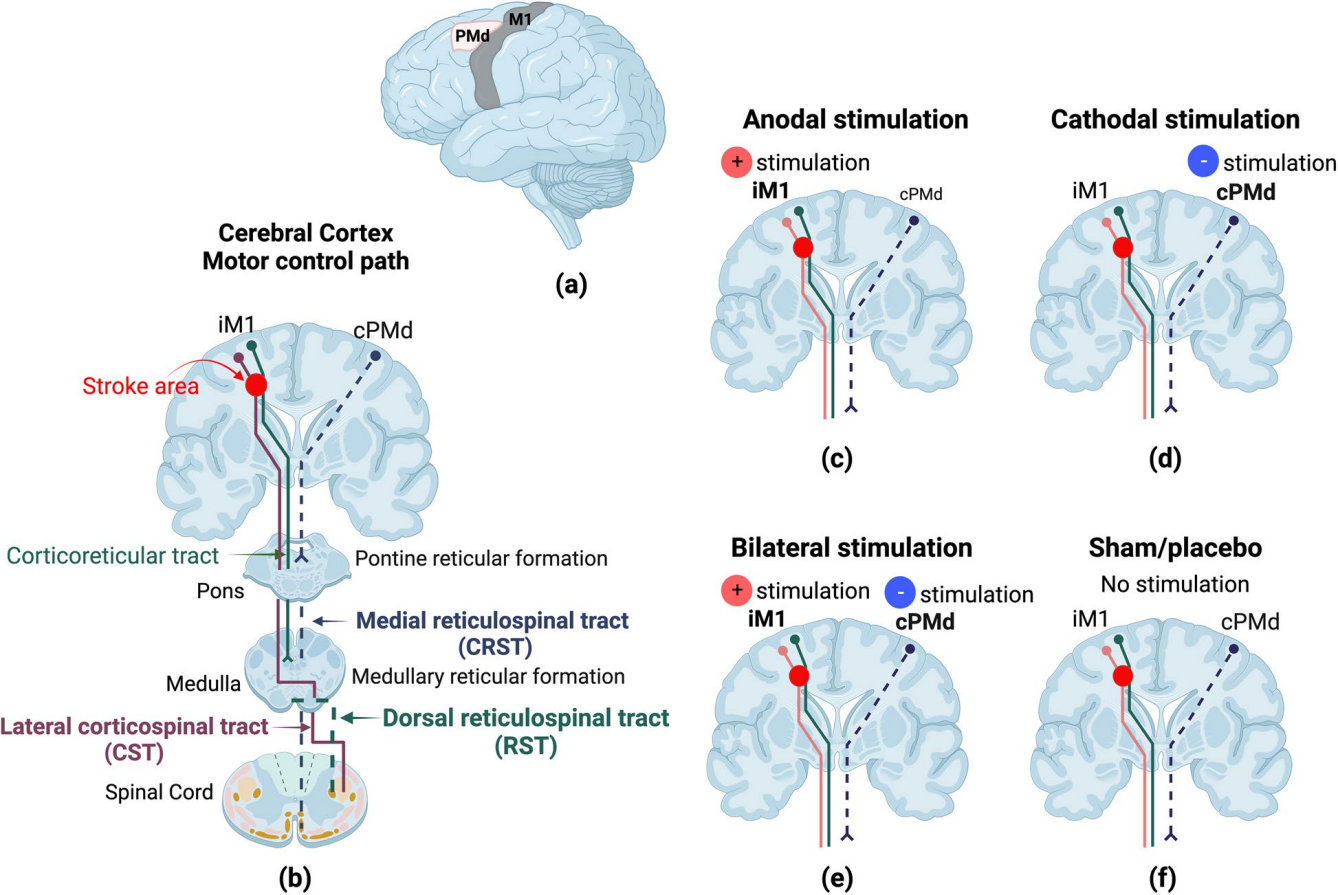
Neural mechanism and the experimental design in this study. **a** The two main motor areas involved in stroke recovery: the primary motor cortex and the dorsal premotor area. **b** Motor descending pathways in stroke recovery. **c** The first intervention involves stimulating the iM1 area using anodal stimulation. **d** The second intervention involves stimulating the cPMd using cathodal stimulation. **e** The third intervention combines anodal stimulation of the iM1 area and cathodal stimulation of the cPMd. **f** The sham condition without any stimulation intervention

Because inhibitory tDCS targeting Wernicke’s area in the right hemisphere has led to improvement in language comprehension in people with global aphasia following left-brain lesions [8], it follows that inhibitory tDCS to the non-lesioned side could diminish interhemispheric inhibition of the lesioned side, aiding in upper extremity motor recovery [8]. Following a stroke, people often exhibit a flexion synergy in the involved upper extremity. When they attempt to elevate their involved shoulder, they experience an involuntary and simultaneous activation of their elbow, wrist, and finger flexors [9]. These involuntary movements are associated with high levels of muscle tone known as spasticity. Prior studies suggest the expression of spasticity is due to recruitment of the reticulospinal tract on the non-lesioned side of the brain, activated to compensate for corticospinal tract (CST) deficits on the lesioned side (Fig. 1b). This hypothesis is based on structural changes and functional connectivity changes occurring after stroke [9, 10]. The purpose of this study is to investigate the impact of facilitating the ipsilesional corticospinal tract via anodal stimulation and inhibiting the contralesional cortico-reticulospinal tract (CRST) via cathodal stimulation (Fig. 1).

## Objectives {7}

This study aims to determine how targeted high-definition transcranial direct current stimulation (THD-tDCS) changes the excitability of the contralesional cortico-reticulospinal tract (CRST) and ipsilesional cortico-spinal tract (CST), thus reducing the expression of the flexion synergy and spasticity.

Specifically, the study aims are as follows:

- Aim one of this study is to evaluate the degree of change of excitability of the CST following anodal Targeted High-Definition transcranial direct current stimulation (THD-tDCS) over the ipsilateral primary motor cortex (iM1) (Fig. 1c). Specifically, we aim to determine whether this stimulation simultaneously reduces contralesional CRST hyperexcitability, while enhancing motor function in the involved upper extremity in participants in the post-acute phase of stroke recovery.
- Aim two is to evaluate the degree of reduction in CRST hyperexcitability as evidenced by a reduction in spasticity and flexion synergy in the involved upper extremity, following cathodal THD-tDCs over the contralesional dorsal premotor cortex (cPMd) in participants in the post-acute phase of stroke recovery (Fig. 1d).
- Aim three combines the first two aims—to evaluate the confluent effect of bilateral THD-tDCS, when given simultaneously using anodal stimulation over iM1 and cathodal stimulation over cPMd (Fig. 1e), on the excitability of CST and CRST, as evidenced by a decrease in the expression of flexion synergy and spasticity, as well as an improvement in UE function.

## Trial design {8}

This trial, conducted at the Carle Foundation Hospital, is an exploratory sham-controlled, double-blind, cross-over study designed to evaluate the effect of THD-tDCS on upper motor function in participants in the post-acute phase of stroke recovery. The study has four intervention arms: anodal stimulation (Fig. 1c), cathodal stimulation (Fig. 1d), bilateral stimulation (both cathodal and anodal) (Fig. 1e), and sham/placebo (Fig. 1f). Participants receive each intervention in a randomized order, with a 2-week washout period between each intervention.

## Methods: participants, interventions, and outcomes

### Study setting {9}

Participants include stroke patients with upper limb motor impairment at the Carle Foundation Hospital. Data will be collected at the Carle Foundation Hospital, Urbana, IL, USA.

### Eligibility criteria {10}

Inclusion criteria are as follows: (i) stroke survivors aged between the ages of 18 and 90 years; (ii) exhibiting paresis confined to one side, experiencing substantial motor impairment of the upper extremity; and (iii) having sufficient cognition to provide informed consent. Exclusion criteria are as follows: (i) muscle abnormalities, motor, or sensory impairment in the *non*paretic upper extremity; (ii) severe atrophy or significant joint contracture; (iii) complete sensory losses in the *paretic* upper extremity or severe cognitive; (iv) affective dysfunction, severe concurrent medical problems, pacemaker, metal implant in the head, or pregnancy; (v) or a known adverse reaction to transcutaneous magnetic stimulation (TMS) or tDCS. All assessment and intervention procedures are conducted by a team of research assistants under the supervision of the principal or co-investigators.

### Who will take informed consent? {26a}

Research personnel obtain informed consent from each participant, explaining the purpose and estimated length of the study, describing the purposes and procedure behind each intervention and each assessment, and discussing each potential risk and benefit. Research personnel then explain how private health information (PHI) is used and the participant’s right to cancel the permission to use or share this information at any time, after which the participant signs a Health Insurance Portability and Accountability act (HIPA) form.

### Additional consent provisions for collection and use of participant data and biological specimens {26b}

N/A. No biological specimens will be collected.

### Interventions

#### Explanation for the choice of comparators {6b}

Participants will be compared to themselves at different visits in order to control for individual factors impacting treatment efficacy. The sham comparator is a mock tDCS session in order to ensure that patients remain blinded to their study arm. In addition, there will be a 2-week wash-out period between visits.

#### Intervention description {11a}

Electrodes are placed into a standard 10–20 EEG cap with a chin strap. Intervention dosage is 2 mA, applied for a duration of 20 min, which constitutes the optimal safe dosage required to influence neuroplasticity according to the safety guidelines of HD-tDCS [11, 12]. Research personnel estimate electrical fields in the brain using the Realistic Volumetric Approach to Simulate Transcranial Electric Stimulation (ROAST) toolbox [13].

#### Criteria for discontinuing or modifying allocated interventions {11b}

Participants have the option to think about the trial before they consent, and to withdraw at any time without any consequences to withdrawal. This study was approved by the Institutional Review Board (IRB) at Carle Foundation Hospital (IRB # 23CNI3891). Written, informed consent and HIPPA authorization are obtained from each participant.

#### Strategies to improve adherence to interventions {11c}

All intervention protocols are performed in one dedicated human subject laboratory housed within the Carle Research Institute. A member of the research team contacts participants to schedule appointments, and telephones participants the day before each appointment. If participants are late to their appointment, they are contacted to determine any need to reschedule. So far, the study has experienced no loss to follow-up, as participants are enthusiastic about the study and eager to return. As part of our effort to promote continued interest in the study, we strive to create a welcoming environment for participants and ensure that they understand they are an integral part of the findings.

#### Relevant concomitant care permitted or prohibited during the trial {11d}

Participants continue their existing health care regimens. Since the pre-intervention and post-intervention assessments are measured on the same day, the participant’s current treatment regimen does not impact on study results, although may impact carry-over between sessions.

#### Provisions for post-trial care {30}

The lead investigators’ phone numbers are provided to participants, and they are advised to contact the team if they experience any side effects. This has not yet occurred. The physician co-investigators are willing to provide care for any participant experiencing adverse effects, although any cost of care may be billed to the participant or the participant’s insurance.

### Outcomes {12}

#### Primary outcome measure

In this study, the primary outcome measure is transcranial magnetic stimulation (TMS) induced motor evoked potentials (MEP) latency and status [14, 15]. These allow the research team to determine the location of the ipsilesional corticospinal tract and the contralesional cortico-reticulospinal tract [14, 16]. Electromyography (EMG) electrodes are applied to the biceps brachii, triceps brachii, brachialis, and deltoid muscles to record muscle response to TMS. Researchers apply the paired-pulse TMS (Magstim® BiStim2, The Magstim Company Ltd., Spring Gardens, Whitland, UK) to the approximate location of the corticospinal tract origin in ipsilesional motor cortex associated with the biceps brachii and the brachialis muscles in the paretic arm. Researchers also apply the TMS to the approximate location of the origin of the cortico-reticulospinal tract utilizing a figure-eight coil [14]. Researchers utilize a paired-pulse TMS with a conditioning pulse at 65% stimulator maximum intensity and follow with a testing pulse at 85% stimulator maximum intensity. This procedure avoids any need to pre-activate the muscle which could cause bias of background EMG [16]. Researchers use paired pulse intervals of 25 ms [14]. Researchers place the center of the coil tangentially to the skull with the handle at 45° from the parasagittal plane: posterior-anterior orientation for ipsilesional M1 and anterior–posterior orientation for contralesional PMd (Fig. 1a) [17, 18]. Researchers define the M1 stimulation location as being on the grid point that results in the largest response in the target muscle. This target is found for the ipsilesional M1 and contralesional M1 hemisphere through stimulation of a 5 × 5 grid of 1 cm spaced sites on the scalp over motor areas of each hemisphere (centered at C3/4 of 10–20 EEG system) [15]. The target of the contralesional PMd is identified using a reference point of 1 cm medial and 2.5 cm anterior of the M1 target at the contralesional hemisphere [17, 19]. Previous research suggests [20] the participant is considered MEP + if MEPs of any amplitude are observed at a consistent latency on at least 5 out 10 trials. If not, the participant is considered MEP − . At least eight additional pulses with an inter-stimulus interval of 2–3 s are applied to the target to obtain an accurate estimate MEP latency. Researchers then calculate the average latency and amplitude of MEP within all positive trials.

#### Secondary outcome measure

The Fugl-Meyer upper extremity assessment (FMA-UE) is a stroke-specific performance-based impairment index with a potential score ranging from 0 to 66, with 66 representing a normal function. The assessment evaluates upper extremity motor function, sensation, passive joint motion, and joint pain and has been shown to have excellent validity and reliability [21, 22]. The FM-UE is often applied in research as an indicator of stroke severity and motor recovery and is considered the “gold standard” against which other measures are compared. The FMA-UE is performed before and after each tDCS session. A subset of the FMA-UE, portion A, is more reflective of the presence of abnormal muscle synergistic activity and is separately calculated as an outcome measure. Previous studies have validated that the minimally clinically significant difference in the FMA-UE assessment is 5 points [21, 22].

#### Other outcome measures

The Modified Ashworth Scale (MAS) grades muscle tone or spasticity on a scale of 0 to 4, with 0 representing no increase in muscle tone and 4 indicating that the muscle causes joint rigidity either in flexion or in extension. The MAS is the primary clinical measure of spasticity following stroke; however, a variety of authors have reported inconsistent inter-rater reliability and validity of the tool with ranges between poor and excellent. The MAS is assessed for the biceps brachii, triceps brachii, anterior deltoid, and flexor digitorum muscles of the fingers before and after each tDCS session [23]. This assessment has been utilized in numerous research studies as a measure of spasticity in patients with upper motor neuron lesions [23].

### Participant timeline {13}

Table 1 displays the timing of the tDCS intervention arms, as well as the timing of each outcome measure. The post-allocation period is repeated at each visit with a different tDCS intervention (anodal, cathodal, sham, or bilateral), for a total of four visits.

**Table 1:** Schedule of enrollment, interventions, and assessments

	**Study period**							
	**Enrolment**	**Allocation**	**Post-allocation**					**Close-out**
**Timepoint**	*** − t***_***1***_	**0**	***t***_***1***_	***t***_***2***_	***t***_***3***_	***t***_***4***_	***t***_***5***_	***t***_***x***_
**Enrollment:**								
**Vitals**	X							
**Informed consent**	X							
**Allocation**		X						
**INTERVENTIONS:**								
***TMS (anodal, cathodal, sham, or bilateral)***				X				
***Washout***							X	
**Assessments:**								
***TMS***			X		X			
***FM-UE***	X		X		X			
***MAS***			X		X			
***Monitor 10–15 min for adverse effects***						X		

### Sample size {14}

Study personnel aim to enroll up to 30 participants (15 female) in the event of a potential attrition rate of 15%. Based on our preliminary data [24], we estimated that the targeted effect size for our study is 0.42 (SD: 0.13). The power analysis was performed by using GLMMPSE Sample Size Software (https://samplesizeshop.org/) [25]. Based on our preliminary data from a pilot trial (ClinicalTrials.gov Identifier: NCT05174949) [24] and the proposed basic statistical analysis on the primary and secondary outcomes (see the “Statistical methods” section), we determined the sample size of 26 will give 80% power at the 5% significance level for the targeted effect size. We will include a total of 30 stroke participants (15 female) for this clinical trial study to account for an attrition rate of 15% based on our previous experience in similar multiple-visit brain stimulation research.

### Recruitment {15}

The primary source of recruitment for this study is the Carle Foundation Hospital. The Carle clinical research team recruit participants who meet inclusion and exclusion criteria based on their medical history. Study personnel then establish an initial visit for these referrals. Other means of referral include word of mouth or Carle Health referrals.

## Assignment of interventions: allocation

### Sequence generation {16a}

The randomization order is also generated by a random sequence generator provided by REDCap (Research Electronic Data Capture) [26, 27].

### Concealment mechanism {16b}

The blinded team members will leave the room when the intervention is performed. The participants are also blinded to the intervention. The interventions for sham, anodal, cathodal, and bilateral stimulation require the same process and length of time and are indistinguishable from the participant’s perspective.

### Implementation {16c}

The randomization order is also generated by a random sequence generator provided by REDCap (Research Electronic Data Capture) [26, 27]. If participants express interest in participating in the study, a sub-investigator will contact them by phone and schedule an appointment. On this visit, research staff will explain the study to them and go through the consent process. After consent is obtained from the participant, the assessments and interventions are performed by trained research staff. The allocation sequence is generated by a member of the team who is not involved in subjective outcome measures including the Fugl-Meyer and MAS.

## Assignment of interventions: blinding

### Who will be blinded {17a}

Trial participants and the team members performing assessments are double-blinded.

### Procedure for unblinding if needed {17b}

The team members performing data analysis are not blinded. There will be no need for unblinding participants and the team members performing assessments, who will not be involved in the data analysis and will not be informed of the order of treatment.

## Data collection and management

### Plans for assessment and collection of outcomes {18a}

A trained clinical investigator who is not involved in the intervention or randomization procedure will perform the assessments. Assessments will be performed at baseline and before and after the intervention at each visit.

### Plans to promote participant retention and complete follow-up {18b}

All intervention protocols are performed in the lab. A member of the research team will contact participants to schedule appointments. Participants receive a phone call the day before their appointment to confirm the meeting. If participants are late to their appointment, they are contacted to see if they are still coming or wish to reschedule. So far, the study has had a high retention rate. Participants are typically enthusiastic about the study and eager to return. As part of our effort to promote continued interest in the study, we strive to create a welcoming environment for participants and ensure that they understand and are included in the process.

### Data management {19}

All participant information, outcome measure recordings, consent forms, and HIPAA forms are stored in the secure data management program, Research Electronic Data Capture (REDCap) [26, 27]. REDCap is a secure, web-based software platform designed to support data capture for research studies, providing (1) an intuitive interface for validated data capture, (2) audit trails for tracking data manipulation and export procedures, (3) automated export procedures for seamless data downloads to common statistical packages, and (4) procedures for data integration and interoperability with external sources.

### Confidentiality {27}

Any paper versions of outcome measures, consent forms, or HIPAA forms are stored in a locked office. The raw/unprocessed data are coded with a unique participant identifier and will be stored in REDCap. Carle Research Institute uses REDCap (Research Electronic Data Capture) software https://redcap.carle.org/REDCap/ for building and managing online surveys and databases. Study data may be stored and accessed via REDCap. REDCap access and maintenance is provided by the Carle Research Institute. REDCap is password-protected. Raw data on the computer cluster is accessible only to study investigators.

### Plans for collection, laboratory evaluation, and storage of biological specimens for genetic or molecular analysis in this trial/future use {33}

N/A. No biological specimens are collected in this study.

## Statistical methods

### Statistical methods for primary and secondary outcomes {20a}

All outcome measures are continuous and correlated in a pretest/posttest format, requiring analysis with longitudinal linear modeling for correlated data analysis. We utilize generalized estimating equations (GEE) in SAS 9.4. GEE analysis is beneficial as it offers an unbiased estimation of population-averaged regression coefficients despite possible misspecification of the correlation structure even when the data is not normally distributed. Intervention type (anodal, cathodal, bilateral, and sham) and the time point (before or after stimulation) are defined as fixed factors. The study participants are defined as random factors. Carry-over effects will be examined using an extension of Grizzle’s classic crossover design [28]. All analyses are conducted using SAS 9.4 (Carey, NC) with an alpha = 0.05.

### Interim analyses {21b}

Interim analyses are performed bi-weekly by study investigators to monitor data quality, completeness, and confirmation of backup. The results of interim analyses are made directly available to the Program Officer at the funding agency on request. No stopping guidelines have been designated due to the low-risk nature of the trial. Any need for trial termination will be determined by the principal investigator.

### Methods for additional analyses (e.g., subgroup analyses) {20b}

The false discovery rate (FDR) correction will be used to decrease the probability of a type I error. All statistical analyses will be performed using SAS/STAT® software.

### Methods in analysis to handle protocol non-adherence and any statistical methods to handle missing data {20c}

If a participant indicates that they no longer wish to participate, they will be removed from the study and will not be included in data analysis.

### Plans to give access to the full protocol, participant-level data, and statistical code {31c}

Any source code developed during this study will be made publicly available through the American Heart Association Precision Medicine Platform. The study protocol and final results can be accessed through ClinicalTrials.gov. There are no plans in place to grant public access to participant-level data.

## Oversight and monitoring

### Composition of the coordinating center and trial steering committee {5d}

The principal investigator will be responsible for monitoring the trial and ensuring participants’ safety throughout the trial. Research staff are responsible for data management. The neurologist who refers participants to the study will be responsible for chart review and diagnosis of medical conditions. There is no patient and public involvement. There is no Trial Steering Committee.

### Composition of the data monitoring committee, its role, and reporting structure {21a}

Data monitoring will be performed by the study investigators and the principal investigators due to the low risk of this study (Risk Determination: Minimal Risk (Approved Categories 1b, 4, 5), Carle Foundation Hospital Intuitional Review Board, August 16, 2023, IRB # 23CNI3819). Raw data will be reviewed biweekly by research personnel to ensure that it is being collected and stored according to the study protocol. Any data quality issues will be reported to the lead investigator.

### Adverse event reporting and harms {22}

Potential adverse effects of tDCS are listed in the consent form and include tingling or warm sensation at the beginning of stimulation, mild, brief headache, redness of the skin under the EMG or intervention electrodes, fatigue, dizziness, or nausea after participating in the experiment. All of these effects are expected to fade soon after the experiment is completed. If participants experience any adverse effects, they report them to the principal investigator, who determines how to proceed. All adverse events are reported to the institutional review board (IRB) as well as the funding agency. The PI holds bi-weekly meetings with team members to review all reportable new information.

### Frequency and plans for auditing trial conduct {23}

Project management group will meet monthly to review trial conduct. The annual report will be made to IRB and the sponsor for reviewing the progress of the trial every year within the trial period.

### Plans for communicating important protocol amendments to relevant parties (e.g., trial participants, ethical committees) {25}

If an amendment is made to the trial protocol it will be reported to the Carle Foundation Hospital IRB and participants will be informed and re-consented before continuing.

## Dissemination plans {31a}

The results of this proof-of-concept trial will be reported in a peer-reviewed journal and presented at national conferences and seminars. In addition, results will be disseminated through ClinicalTrials.gov, in accordance with institutional policies that ensure compliance with AHA policies on clinical trial registration and reporting. Data will be available upon request after the results of the trial are accepted for publication.

## Discussion

This study will add to a growing body of research regarding the use of targeted high-definition tDCS to aid in the recovery of motor function for stroke participants. Specifically, this study will compare the role of the contralesional premotor cortex and corticoreticulospinal tract to that of the ipsilesional primary motor cortex and corticospinal tract in post-stroke motor recovery. The effectiveness of anodal stimulation over the ipsilesional primary motor cortex is well-documented, but the benefit of adding cathodal stimulation to the contralesional side is more controversial [8]. This study will add to our knowledge of how targeted high-definition tDCS, especially the cathodal stimulation targeting contralesional dorsal premotor cortex and bilateral stimulation, might be useful as part of a treatment for stroke survivors and may support the explanation that the mechanism by which the excitability change of contralesional dorsal premotor cortex is related to the expression of CRST hyperexcitability and motor impairments post-stroke.

## Trial status

Protocol version: 23 July 2022, Version n. 1

Date of recruitment: 1 Oct 2022.

End of recruitment: estimated to be June 2025.

## Data Availability

The study investigators have access to the final trial dataset. The datasets used and/or analyzed during the current study are available from the corresponding author upon reasonable request.

## Abbreviations

THD-tDCS: Targeted high-definition transcranial direct current stimulation
CRST: Corticoreticulospinal tract
CST: Corticospinal tract
cPMd: Contralesional dorsal premotor cortex
iM1: Ipsilesional primary motor cortex
MAS: Modified Ashworth Scale
MEP: Motor evoked potential
TMS: Transcranial magnetic stimulation
EMG: Electromyography
